# Effects of 3β-Acethyl Tormentic Acid (3ATA) on ABCC Proteins Activity

**DOI:** 10.3390/ijms13066757

**Published:** 2012-06-04

**Authors:** Gleice da Graça Rocha, Marisol Simões, Rodrigo Rodrigues Oliveira, Maria Auxiliadora Coelho Kaplan, Cerli Rocha Gattass

**Affiliations:** 1 Laboratory of Cellular Immunology, Carlos Chagas Filho Institute of Biophysics, Federal University of Rio de Janeiro, Rio de Janeiro 21949-900, RJ, Brazil; E-Mails: gleicegr@gmail.com (G.G.R.); marisol.simoes@bio.fiocruz.br (M.S.); 2 Department of Natural Products Research, Federal University of Rio de Janeiro, Rio de Janeiro 21949-900, RJ, Brazil; E-Mails: roroliveira@uenf.br (R.R.O.); makaplan@hotmail.com (M.A.C.K.)

**Keywords:** ABCC transporter inhibitor, multidrug resistance, ABC transporters, 3β-acetyl tormentic acid, triterpenes

## Abstract

Multidrug resistance (MDR) is considered the main cause of cancer chemotherapy failure and patient relapse. The active drug efflux mediated by transporter proteins of the ABC (ATP-binding cassette) family is the most investigated mechanism leading to MDR. With the aim of inhibiting this transport and circumventing MDR, a great amount of work has been dedicated to identifying pharmacological inhibitors of specific ABC transporters. We recently showed that 3β-acetyl tormentic acid (3ATA) had no effect on P-gp/ABCB1 activity. Herein, we show that 3ATA strongly inhibited the activity of MRP1/ABCC1. In the B16/F10 and Ma104 cell lines, this effect was either 20X higher or similar to that observed with MK571, respectively. Nevertheless, the low inhibitory effect of 3ATA on A549, a cell line that expresses MRP1-5, suggests that it may not inhibit other MRPs. The use of cells transfected with ABCC2, ABCC3 or ABCC4 showed that 3ATA was also able to modulate these transporters, though with an inhibition ratio lower than that observed for MRP1/ABCC1. These data point to 3ATA as a new ABCC inhibitor and call attention to its potential use as a tool to investigate the function of MRP/ABCC proteins or as a co-adjuvant in the treatment of MDR tumors.

## 1. Introduction

Multidrug resistance (MDR) is a significant problem in the treatment of many types of cancer. In the clinical setting, patients that exhibit overexpression of MDR proteins within tumors are usually not responsive to a broad spectrum of structurally and functionally unrelated anticancer agents. MDR can be conferred by many mechanisms. One of the best studied of these mechanisms is the active drug efflux mediated by transporter proteins of the ABC (ATP-binding cassette) family. By actively removing cytotoxic drugs from cancer cells, these proteins decreased intracellular drug accumulation below toxic levels, hence preventing cell death [1]. Among the 48 human ABC transporters described to date, at least 12 have been suggested to play a role in MDR [2]. Of these transporters, the most clinically relevant are P-glycoprotein (P-gp/ABCB1), multidrug resistance protein 1 (MRP1/ABCC1) and breast cancer resistance protein (BCRP/ABCG2). These transporters have been shown to confer resistance in both *in vitro* and *in vivo* model systems, and their expression has been correlated with a poor prognosis [3].

The ABCC branch is the largest among the ABC family of transporters, including 12 proteins, nine of which are often referred to as multidrug resistance proteins (MRP1-9). Several studies have shown that these proteins present from 33 to 58% amino acid homology and are able to transport a large range of endogenous and xenobiotic anionic substances across the plasma membrane. Among these proteins, MRP6 (ABCC6) and MRP9 (ABCC12) are not known to transport drugs, and the substrates of MRP5 and MRP7 remain unknown. Thus, the functions, substrates and cancer significance of MRP1-MRP4 are the best known among the proteins of the ABCC subfamily [4,5].

The clinical relevance of ABCC proteins in cancer resistance has been investigated since the characterization of MRP1 (ABCC1). In tumor cells, the 190 kDa MRP1 protein can confer resistance to a large number of antineoplastic drugs, including anthraciclines, such as doxorubicin and daunorubicin, vinca alkaloids, such as vincristine, and other drugs, including etoposide and methotrexate (MTX) [6]. Moreover, this protein is also able to transport some of the newer so-called ‘targeted’ agents (e.g., certain tyrosine kinase inhibitors) that modify various signal transduction pathways [7]. Clinically, overexpression of MRP1 has been detected in a variety of tumor types and has been associated with drug resistance or poor patient outcomes related to a variety of tumor types, including lung carcinoma [8], breast carcinoma [9], gastric carcinoma [10] and neuroblastoma [11].

A role of other ABCC proteins (MRP2–4) in cancer resistance has also been demonstrated. *In vitro* studies have found that MRP2 transports a variety of anticancer drugs, including MTX, cisplatin, irinotecan, paclitaxel and vincristine [2]. Expression of this protein is detected in some solid tumors originating from the colon, gastric and lung [12] as well as in cells from patients with acute myelogenous leukemia [13] and esophageal carcinoma [14]. Recently, resistance in patients with hepatocellular carcinoma treated with cisplatin-based chemotherapy was also attributed to MRP2 expression [15].

Although MRP3/ABCC3 transports fewer anticancer substrates than MRP1 [16], overexpression of MRP3 has been predicted to be a prognostic factor in childhood and adult acute lymphoblastic leukemia and adult acute myeloid leukemia [13] and pancreatic carcinoma [17]. In addition, elevation of the levels of MRP3 expression has been detected in human hepatocellular carcinomas [18], non-small cell lung cancer [19] and primary ovarian cancer [20].

MRP4/ABCC4 is able to transport several eicosanoids, including prostaglandin E2, that have implications for tumor development, growth, and angiogenesis and the response of tumors to cytotoxic chemotherapy [21]. This protein has a remarkable ability to transport methotrexate (MTX), and 6-mercaptopurine (6-MP), and thiopurine [16], and it has been implicated in the highly proliferative growth of some tumors, including prostate tumors and neuroblastomas [11,22].

**Figure 1:**
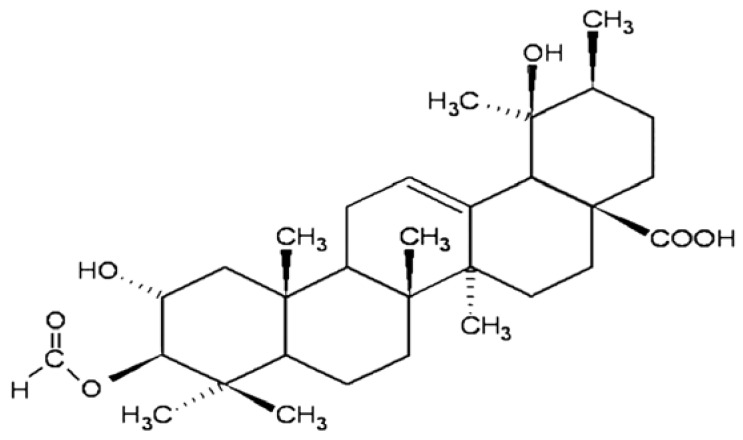
Structure of 3β-acetyl tormentic acid.

Successful treatment of cancer requires not only anticancer drugs but also the potential to inhibit MDR in cancer cells. The observation that many compounds of natural origin are capable of modulating MDR proteins has called attention to these substances. Pentacyclic triterpenes have recently been emerging as a class of antitumoral substances with anti-MDR properties [23–26]. In a previous study [27], we showed that 3β-acetyl tormentic acid (3ATA, Figure 1), a triterpene isolated from *Cecropia lyratiloba*, is able to induce apoptosis in an MDR leukemia cell line overexpressing P-gp/ABCB1 without interfering with P-gp/ABCB1 expression or activity. The aim of the present work was to study the interactions of 3ATA with members of the MRP/ABCC family and, thus, to build a profile of the activity of this compound with respect to this transporter family. We showed that 3ATA strongly inhibited pump activity in murine melanoma B16F10 and monkey epithelial Ma104 cell lines expressing the MRP1/ABCC1 protein. However, 3ATA showed a weaker effect in the A549 a lung adenocarcinoma cell line which expresses various members of the MRP/ABCC family, suggesting that this triterpene may exhibit a higher selectivity for MRP1. Indeed, even though 3ATA was able to downmodulate the activity of the other MRPs, the inhibition ratio of MRP1/ABCC1 was significantly higher than that of MRP2/ABCC2 [28], MRP3/ABCC3 [29] or MRP4/ABCC4 [30]. Taken together, our data indicate 3ATA as an interesting drug for use in investigating the function and activity of MRP/ABCC proteins. Our findings also call attention to the potential of this compound as a co-adjuvant for the treatment of tumors in which resistance is mediated by MRP/ABCC proteins.

## 2. Results and Discussion

### 2.1. Results

#### 2.1.1. Cytotoxicity of 3ATA to MRP-1/ABCC1-Expressing Cell Lines

**Figure 2:**
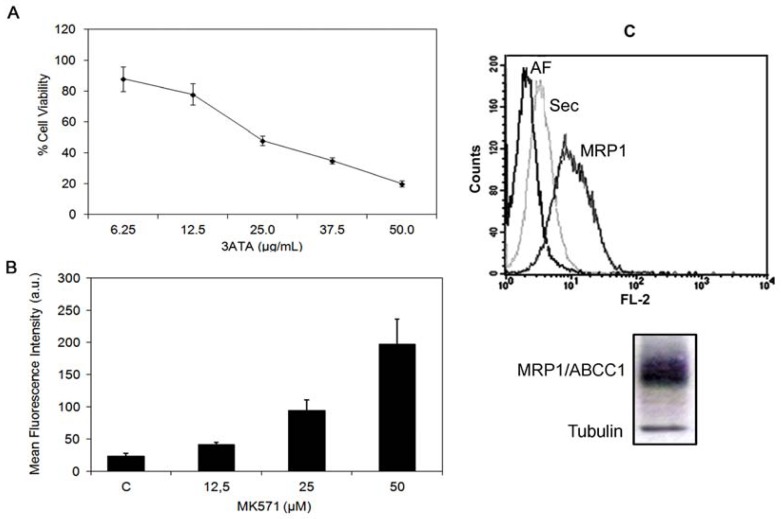
3ATA decreases the viability of the B16/F10, a cell line that expresses active MRP1/ABCC1. (**A**) The effects of 3ATA on the viability of B16F10 cells were assessed by MTT assays as described in the Experimental Section (ES). The results, presented as the percentage of cell viability, represent the mean ± SD of 2 experiments performed in triplicate. (**B**) The activity of MRP1/ABCC1 in B16/F10 cells was measured using flow cytometry in cells incubated with 5 μM CFDA in the absence (control) or presence of different concentrations of MK-571, a conventional MRP1 inhibitor, as described in ES. The results are presented as arbitrary units (a.u.) of the mean fluoresce intensity from 3 experiments. (**C**) The expression of MRP1/ABCC1 was evaluated by flow cytometry (top) and western blotting (bottom) as described in ES. (Top) Histogram showing the fluorescence of B16F10 cells treated with medium (AF), PE-labeled secondary antibody (sec) or MRP1/ABCC1 antibody (MRP1). (Bottom) Western blotting analysis of B16F10 cells extract revealed using tubulin and MRP1/ABCC1 antibodies.

In a previous study, we showed that 3ATA induced apoptosis in P-gp/ABCB1-expressing cell lines without interfering with pump activity [27]. Herein, we used the MTT assay to evaluate the cytotoxicity of this triterpene against MRP1/ABCC1-expressing cell lines. Considering the expression and the role of MRP1 in drug resistance in human melanomas, we used B16F10, a murine melanoma cell line that expresses high levels of active MRP1 (Figure 2B,C). The results showed that 3ATA decreased the viability of the cells in dose-dependent manner (Figure 2A). Treatment with 25 μg/mL of 3ATA was able to inhibit the viability of approximately 50% of the cells.

**Figure 3:**
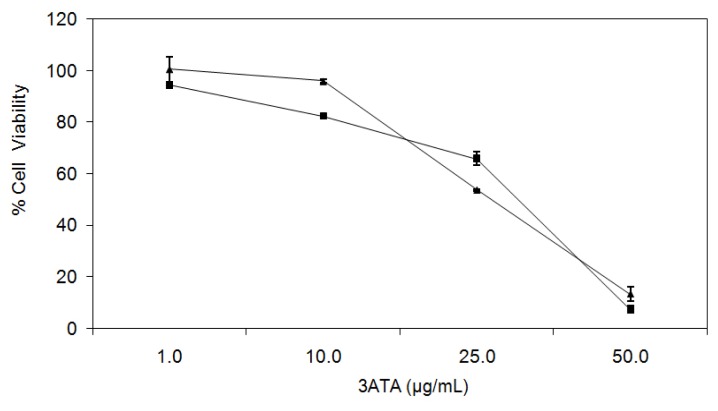
3ATA decreases the viability of MRP1/ABCC1-expressing cell lines, Ma104 and A549. Effects of 3ATA on the viability of Ma104 (square) and A549 (triangle) cells were assessed by MTT assay, as described in ES. The results, presented as the percentage of cell viability, represent the mean ± SD of 3 experiments performed in triplicate.

To confirm these observations, we assessed the effect of 3ATA in Ma104, an epithelial (monkey kidney embryo) non-cancer cell line that expressed both P-gp/ABCB1 and active MRP1 [31], and in A549, a human lung adenocarcinoma cell line that expresses several members of the MRP/ABCC subfamily [32]. The obtained results (Figure 3) confirmed the ability of 3ATA to inhibit the viability of cell lines expressing MRP1.

#### 2.1.2. Modulation of MRP1/ABCC1 Activity by 3ATA

**Figure 4:**
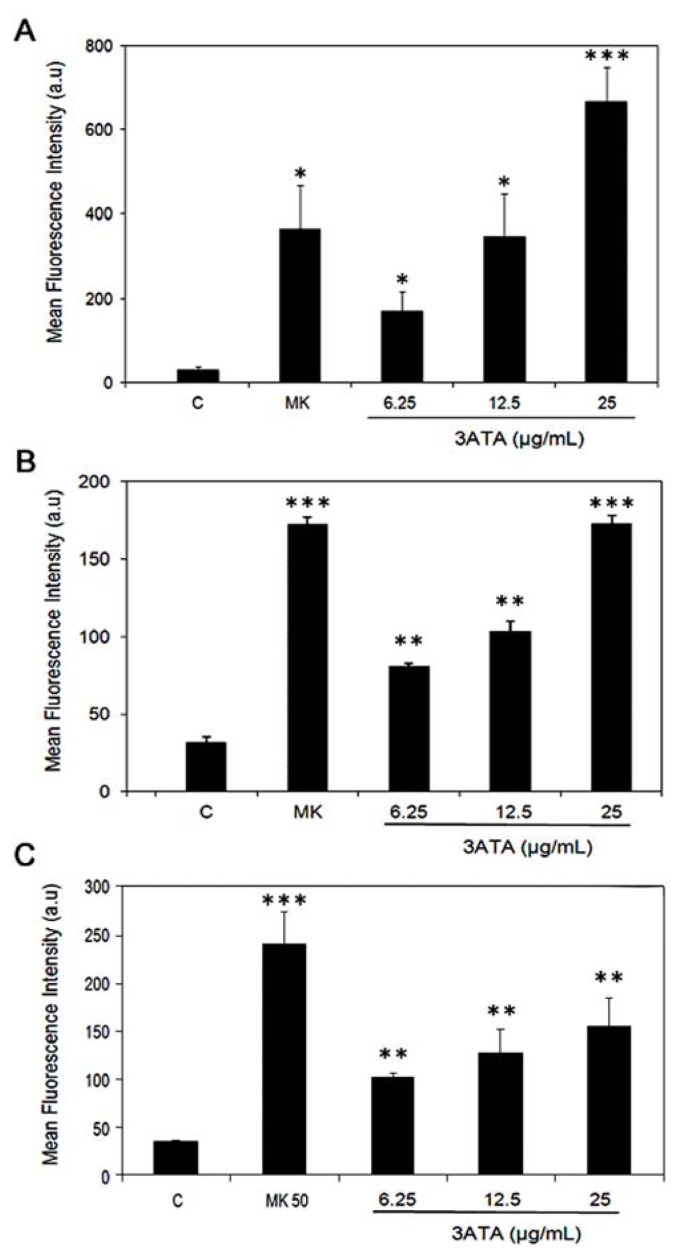
Effects of 3ATA on the activity of MRP1/ABCC1-expressing cell lines. The activity of the transporter proteins was evaluated as described in ES by measuring the accumulation of a fluorescence probe. (**A**) B16/F10, (**B**) Ma104 and (**C**) A549 cells were incubated with medium or 5 μM CFDA in the absence (control) or presence of 50 μM MK-571 (MK) or different concentrations of 3ATA, and cell fluorescence was measured by flow cytometry. The results are expressed as the mean ± SD of the mean fluorescence intensity (MIF) obtained in 3 different experiments. *, ** and *** indicated *p* < 0.01, *p* < 0.005 and *p* < 0.001, respectively, in relation to the control.

To continue our analysis of the effect of 3ATA on ABC transporters, we examined whether 3ATA is able to modulate the pump function of MRP1/ABCC1. Several studies have shown that cells exhibiting high levels of the ABCC1 protein actively exclude carboxy-fluorescein (CF), a product of the cleavage of 5-CFDA by cellular esterases which can therefore be used as an indicator of MRP1 pump activity [31]. The Mean Fluorescence Intensity (MFI) associated with intracellular CF, which reflects the efflux activity of MRP1, was used to quantify this activity. To investigate the effects of 3ATA on MRP1 transport, cells (B16F10, Ma104 or A549) were loaded with 5-CFDA in the presence of medium, MK571 (a commercial inhibitor of MRP1) or different concentrations (6.25, 12.5 or 25 μg/mL) of 3ATA, and intracellular fluorescence was evaluated. Treatment with 3ATA inhibited the activity of MRP1 in the cell lines in a dose-dependent manner. However, the degree of inhibition varied among different cell lines. In fact, at the highest dose of this triterpene (25 μg/mL), the fluorescence of the dye retained in B16F10 cells was 20 times higher than in the control and was even higher than in MK571-treated cells (Figure 4A). In Ma104 cells, the inhibitory effect of 25 μg/mL 3ATA (Figure 4B) on MRP1 activity was very similar to that of MK571, while in A549 cells, the inhibitory activity of this triterpene was surprisingly low (Figure 4C). These results show that 3ATA strongly inhibited MRP1/ABCC1 activity in cells expressing this transporter. Because A549 cells also express other MRPs, it is possible that the low effect of 3ATA in this cell line was due to transport mediated by MRPs other than MRP1.

#### 2.1.3. Effects of 3ATA on the Activity of Other MRP/ABCC Transporter Proteins

**Figure 5:**
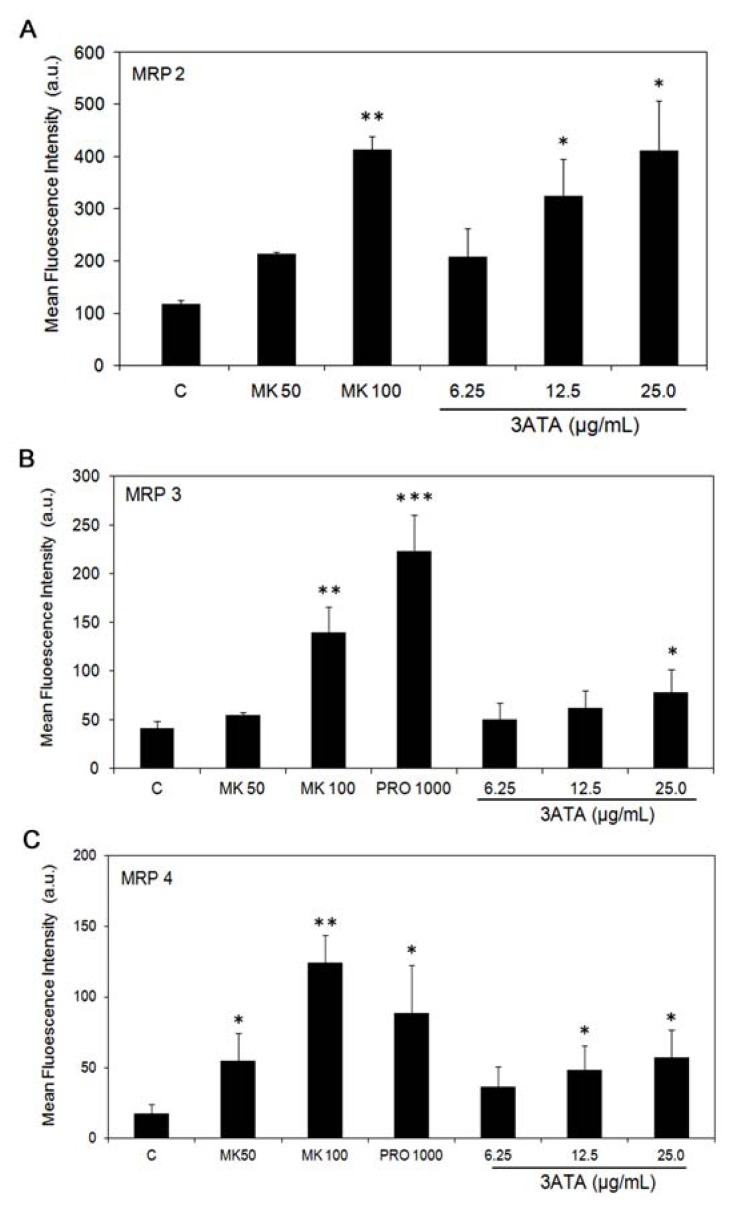
Effect of 3ATA on MRP2/ABCC2, MRP3/ABCC3 and MRP4/ABCC4 activity. Determination of MRP2 activity in 2008/MRP2 cells (**A**), MRP3 activity in HEK/MRP3 cells (**B**) and MRP4 activity in 3T3/MRP4 cells (**C**) was performed essentially as described in Figure 3, except that MK571 was used at concentrations of 50 μM and 100 μM, and probenicid, another MRP1 inhibitor, was used at 1,000 μM. The results are expressed as the mean ± SD of the mean fluorescence intensity obtained in 3 different experiments. *, ** and *** indicated *p* < 0.05, *p* < 0.005 and *p* < 0.0005, respectively, in relation to the control.

To investigate the hypothesis that the inhibitory effect of 3ATA is more selective for MRP1/ABCC1 compared to other MRPs, we used cell lines that overexpressed MRP2/ABCC2 (2008/MRP2) [28], MRP3/ABCC3 (HEK/MRP3) [29] or MRP4/ABCC4 (3T3MRP4) [30]. As shown in Figure 5A–C, at 25 μg/mL, 3ATA was able to modulate the activity of all ABC transporters that were assessed.

In order to compare the inhibitory effects of 3ATA on the different MRPs/ABCCs, we used data from Figures 3 and 4 and calculated an inhibition ratio dividing the mean fluorescence intensity (MIF) of treated cells by the MIF of untreated ones. As can be seen in Table 1, the inhibitory potential of 3ATA against MRP2-4/ABCC2-4 proteins is much lower than that observed for MRP1/ABCC1.

### 2.2. Discussion

Multidrug resistance caused by the overexpression of ABC drug transporters is a major obstacle in modern cancer chemotherapy. In the last decade, considerable attention has been dedicated to the role played by these transporters and to the search for new approaches to overcome the mechanisms of drug resistance. However, finding a selective, low toxicity inhibitor/modulator of ABC drug transporters represents a complex challenge. As ABC proteins are present not only in cancer tissues but also in normal tissues, the specificity, potency and intrinsic toxicity of an ideal inhibitor should be associated with an ability to preserve the physiological functions of the MDR proteins in normal tissues [33].

The first inhibitors/modulators of P-glycoprotein (P-gp/ABCB1) were discovered in the beginning of the 1980s. Since then, numerous inhibitors of this protein have been described, many of which are presently being tested in clinical trials, with some promising results having been obtained. As MRP1/ABCC1 was discovered much later, the first inhibitors of this protein, including difloxacin, a quinolone antimicrobial agent [34], the competitive inhibitor of ATP-dependent LTC4 transport MK571 [35] and the non-specific inhibitor of organic anion transport probenecid, were identified only in second half of the 1990s [34] Several cyclohexyl-linked tricyclic isoxazoles have also been described as potent and specific inhibitors of MRP1/ABCC1 [36]. Furthermore, some substances that are able to inhibit MRP1/ABCC1, such as Biricodar (VX-710), Elacridar and CBT-1, were originally developed as P-gp/ABCB1 inhibitors [4,37]. However, the number of selective modulators of MRP1/ABCC1 is smaller than for P-pg, and none of them have been extensively investigated in clinical trials.

The search for compounds that are able to modulate these proteins is still poorly developed. Among the few substances that can modulate other members of the MRP/ABCC subfamily, most also inhibit MRP1/ABCC1 activity. These include probenecid, a general ABCC inhibitor [38]; MK571, which in addition to MRP1/ABCC1 [35], also modulates MRP2/ABCC2 [39], MRP4/ABCC4 [40], MRP3/ABCC3 and MRP5/ABCC5 [37]; and Indomethacin and dietary flavonoids, which also inhibit MRP4/ABCC4 [4,41] and MRP2/ABCC2 [38], respectively, in addition to MRP1/ABCC1. With respect to MRP3/ABCC3, the few substances reported to be able to inhibit this protein include non-nucleoside reverse transcriptase and nucleoside reverse transcriptase inhibitors that also inhibit MRP1/ABCC1 and MRP4/ABCC4 [37].

Despite the great efforts dedicated to the development and identification of MDR inhibitors, none have proven to be without side effects, and the problem of circumventing MDR remains a challenge. Recently, many researchers have turned their attention to natural products as a source of substances for circumventing MDR mediated by ABC transporters in cancer treatment. Some of these compounds bypass MDR because they are not substrates for these pumps [23,42]. Others work as modulators of the transporter proteins [43–45].

We investigated the effect of triterpenes isolated from *Cecropia lyratiloba* on cells expressing MDR proteins and demonstrated that these triterpenes are not substrates for the P-gp/ABCB1 protein [27]. This study shows that 3ATA (3β-acetyl tormentic acid), one of the triterpenes isolated from this plant, is able to kill MRP1/ABCC1-expressing cells (Figures 2A and 3). However, in contrast to its effect on P-gp/ABCB1-expressing cells, data presented in this paper clearly show that 3ATA strongly inhibits the activity of the MRP1/ABCC1 protein. In one of the investigated cell lines (B16F10), the inhibitory power of 3ATA is even higher than that of a commercial inhibitor of this protein, MK571 (Figure 4A) [35], whereas in another cell line (Ma104), its inhibitory effect is similar to that of MK571 (Figure 4B). The results, showing a low inhibitory effect of 3ATA in a cell line that expresses several MRPs [32] and active MRP3/ABCC3 [19], suggested that this triterpene could be a specific inhibitor of MRP1/ABCC1. However, the use of cell lines expressing MRP2/ABCC2, MRP3/ABCC3 or MRP4/ABCC4 revealed that 3ATA was also able to inhibit these transporters (Figure 5A–C), although the associated inhibition ratio values are lower than that obtained for MRP1/ABCC1 (Table 1). These results may reflect a difference in the specificity of these transporters with respect to 3ATA and suggest that at the concentrations of 3ATA used the activity of MRP2–4 is only partially inhibited. Indeed, at higher concentrations 3ATA (75–100 μg/mL) the accumulation of CF is very similar to that observed with MK571 (results not shown).

Because the presence of other ABC transporters in tumors can complicate the use of specific modulators of ABC transporters, the use of non-specific modulators is a still viable approach for treating drug-resistant cancers. Furthermore, due to its ability to inhibit the pump activity of ABCC proteins, 3ATA may represent an interesting tool for investigating the structure-function relationships of the efflux pumps involved in the transport mechanism and drug selectivity of these proteins and to aid in understanding their physiological functions and substrates. Considering the transport activity of the ABCC subfamily of proteins in the kidneys, liver, intestinal epithelia and the blood brain barrier [5], it is possible that by modulating this transport activity, 3ATA would increase the bioavailability of drugs and therefore be useful as a co-adjuvant in cancer treatment. Taken together, our results indicate that 3ATA is a novel and potent ABCC subfamily modulator with greater selectivity for MRP1 and may be considered as a promising lead compound for the design of more efficient multidrug resistance chemosensitizers or reversal agents.

## 3. Experimental Section

### 3.1. Chemicals and Cell Culture

3β-acetyl tormentic acid (MW 531.71), isolated from *Cecropia lyratiloba* as described previously [42], was dissolved in dimethyl sulfoxide (DMSO, SIGMA, St. Louis, MO, USA) and diluted in culture media. Probenecid (Kindly donated by Dr. V.M.B.D. Rumjanek, Federal University of Rio de Janeiro, Brazil), MK-571 and a monoclonal antibody against MRP1/ABCC1 (clone A23) and Tubulin (clone b-7, 1:500) were obtained from Enzo Life Sciences (Farmingdale, NY, USA) and Santa Cruz Biotechnology (Santa Cruz, CA, USA). The B16F10 murine melanoma, A549 human lung adenocarcinoma, Ma104 epithelial (monkey kidney embryo), 2008/MRP2 ABCC2—transfected human ovarian carcinoma [28], HEK/MRP3 ABCC3-transfected HEK (human embryonic kidney 293) [29] and 3T3MRP4 ABCC4-transfected NIH3T3 (mouse embryonic fibroblast) [30] cell lines were cultured at 37 °C under 5% CO_2_ in the recommended medium (DMEM or RPMI 1640 (Life Technologies, Inc., Rockville, MD, USA) supplemented with heat-inactivated 10% fetal calf serum (FCS, Life Technologies, Inc., Rockville, MD, USA), 2 mM L-glutamine, 100 U/mL of penicillin and 100 mg/mL of streptomycin (Life Technologies, Inc., Rockville, MD, USA). The HEK/MRP3 and 3T3MRP4 cells lines were kindly donated by Drs. Elizabeth Hopper-Borge (Assistant professor) from the Fox Chase Cancer Center, Buckingham, PA, USA. The 2008/MRP2 cell line and its parental cell lines were purchased from the Stichting Het Nederlands Kanker Instituut (NKI-AVL), Amsterdam, the Netherlands.

### 3.2. MTT Assay

Drug cytotoxicity was assessed using the MTT assay as described previously [23]. For these assays, 180 μL of cell suspension (10^4^ per well) was distributed in 96-well plates and pre-incubated for 24 h at 37 °C/5% CO_2_ to allow stabilization of the culture. Cells were exposed to 20 μL of medium, different concentrations of 3ATA (1.0, 6.25, 10.0, 12.5, 25.0, 50.0 μg/mL or 1.88, 11.76, 18.81, 23.51, 47.02, 94.04 μM) or DMSO (at the same concentration carried by the compounds as a control). After 48 h of incubation, the cultures were treated with 20 μL of MTT (5 mg/mL) and held for 4 h at 37 °C before being centrifuged, after which the supernatant was discarded. The formazan produced via reduction of MTT by viable cells was dissolved in DMSO, and the optical density was measured in an ELISA reader (BenchMark, Bio-Rad, Hercules, CA, USA) at 570 nm (reference 630 nm). All determinations were carried out in triplicate, and the average standard error was always <5%. The IC_50_ values were calculated from concentration-response curves through linear regression analysis.

### 3.3. Activity and Expression of MRP/ABCC Proteins

The activity of MDR proteins was determined based on the accumulation of specific substrates. To measure the activity of the MRP subfamily members, 5-carboxyfuorescein diacetate (CFDA), a non-fluorescent molecule that is converted into fluorescent carboxy-fluorescein (CF) by intracellular esterases, was used [31]. For each experiment, cells (1 × 10^5^/well) were seeded into 24-well plates and pre-incubated for 24 h at 37 °C/5% CO_2_ to allow stabilization of the culture. Cells were then incubated for 30 min with medium (autofluorescence), or with 5 μM CFDA in the presence of medium, inhibitors of MRPs (50 and 100 μM MK-571 or 1000 μM Probenecid) or the desired concentrations of 3ATA. The cells were subsequently washed in PBS, harvested and kept on ice until flow cytometry analysis was performed (FACSCalibur, Beckton-Dickinson cytometer). The results are presented as representative histograms or as the mean ± SD of arbitrary units of mean fluorescence intensity (MFI).

Evaluation of MRP1 expression in B16/F10 cells was performed by flow cytometry or western blotting. For flow cytometry analysis, cells were harvested, permeabilized with FACS lysing solution for 30 min and incubated for 10 min with a blocking solution (PBS with 5% BFS). The cells were then centrifuged (1400 rpm/5 min) and resuspended in PBS solution with an anti-MRP1 antibody (1:50 dilution, clone A23) for 60 min at room temperature. After two washes with PBS, the cells were incubated with a PE-labeled goat anti-rat IgG antibody (1:5000) from Sigma for 30 min. After washing with PBS, the cells were resuspended in PBS, and their fluorescence was evaluated by flow cytometry (FACSCalibur, Beckton-Dickinson cytometer, FL-2). The results are presented as representative histograms. For western blotting analysis, whole-cell extracts were prepared by diluting the cell pellets directly in RIPA [50 mM Tris-HCl, pH 8.0, 150 mM NaCl, 0.5% sodium deoxycholate, 0.1% SDS and 1% protease inhibitor (Amersham, Arlington, IL, USA)]. Equal amounts of protein were separated by SDS-PAGE and transferred to polyvinylidene difluoride (PDF) membranes. The blots were blocked for 2 h in PBS/0.05% Tween containing 5% nonfat dried milk, probed with specific antibodies to MRP1 (clone A23, 1:1000) or Tubulin (clone b-7, 1:500) overnight and incubated with HPR-conjugated secondary antibodies (1:2000 dilution; Amersham Biosciences, Buckinghamshire, UK) for 1 hour at room temperature. The blots were developed using an enhanced chemiluminescence system (ECL, Amersham, Arlington, IL, USA) according to the manufacturer’s instructions.

### 3.4. Statistical Analysis

The data from three independent experiments are expressed according to the mean and standard deviation (SD). Differences were assessed using the unpaired Student’s *t*-test (Software GraphPad Instat 3.1, San Diego, CA, 1998).

## 4. Conclusion

The triterpene 3β-acetyl tormentic acid is an inhibitor of the ABCC subfamily of transporters with greater selectivity for MRP1/ABCC1. This triterpene can be used as a tool to investigate the function and activity of MRP/ABCC proteins. It probably can also be used for the design of multidrug resistance chemosensitizers or reversal agents.

## Figures and Tables

**Table 1:** Comparative inhibitory effects of 3ATA on the activity of MRP/ABCC proteins. The inhibitory ratios were obtained from data of Figures 3 and 4 by dividing the mean fluorescence intensity (MFI) of treated cells by the MFI of untreated cells. Results as expressed as mean ± SD. of data from 3 different experiments.

Inhibition Ratio						

Treatment	B16F10	Ma104	A549	2008/MRP2	HEK/MRP3	3T3/MRP4
MK50	10.5 ± 1.7	5.4 ± 0.3	7.0 ± 1.1	1.8 ± 0.3	1.5 ± 0.2	3.7 ± 0.7
MK100	-	-	-	3.6 ± 0.3	3.7 ± 0.1	6.1 ± 1.4
PRO 1000	-	-	-	-	6.0 ± 0.1	7.7 ± 1.3
3ATA 6.25	5.6 ± 1.2	2.5 ± 0.1	2.9 ± 0.2	1.8 ± 0.2	1.2 ± 0.3	2.3 ± 0.4
3ATA 12.5	11.4 ± 1.3	3.2 ± 0.1	3.0 ± 0.3	2.7 ± 0.1	1.4 ± 0.3	3.1 ± 0.5
3ATA 25.0	20.9 ± 3.4	5.4 ± 0.2	4.0 ± 0.4	3.5 ± 0.2	1.7 ± 0.4	3.5 ± 0.3

- not determined.
